# ‘Medical clearance’ and referral to liaison psychiatry: a national service evaluation

**DOI:** 10.1192/bjb.2023.43

**Published:** 2024-06

**Authors:** George Gillett, Sophie Westwood, Alex B. Thomson, William Lee

**Affiliations:** 1 Institute of Psychiatry, Psychology and Neuroscience, King's College London, London, UK; 2 Department of Psychiatry, University of Oxford, Oxford, UK; 3 University of Plymouth, Plymouth, UK; 4 Central and North West London NHS Foundation Trust, London, UK; 5 Cornwall Partnership NHS Foundation Trust, Bodmin, UK

**Keywords:** Liaison psychiatry, medical clearance, qualitative research, clinical governance, comorbidity

## Abstract

**Aims and method:**

The prevalence of delaying psychiatric care until the patient has received ‘medical clearance’, and the definitions and understanding of ‘medical clearance’ terminology by relevant clinicians, are largely unknown. In a service evaluation of adult liaison psychiatry services across England, we explore the prevalence, definitions and understanding of ‘medical clearance’ terminology in three parallel studies: (a) an analysis of trust policies, (b) a survey of liaison psychiatry services and (c) a survey of referring junior doctors. Content and thematic analyses were performed.

**Results:**

**‘**Medical clearance’ terminology was used in the majority of trust policies, reported as a referral criterion by many liaison psychiatry services and had been encountered by most referring doctors. ‘Medical clearance’ was identified as a common barrier to liaison psychiatry referral. Terms were inconsistently used and poorly defined.

**Clinical implications:**

Many liaison psychiatry services seem not to comply with guidance promoting parallel assessment. This may affect parity of physical and mental healthcare provision.

There is much comorbidity between mental and physical health conditions^1^ and there is also a relationship between mental illness and general hospital admission for physical illness.^2,3^ Consequently, many medical in-patients require liaison psychiatry input.^4^ Patients attending an emergency department with primarily psychiatric presentations often require medical investigations alongside psychiatric assessment.^5^ The recommended service model is to deliver liaison psychiatry care side by side with medical care.^6–8^

Despite this, medical care and psychiatric care are often provided sequentially rather than simultaneously. The prevalence of delaying psychiatric assessment until the patient has received ‘medical clearance’ is largely unknown.^9^ We believe that several terms related to the concept of ‘medical clearance’, such as ‘medically fit for discharge/assessment’, are used in practice. Such terms may confer different meanings in different contexts.^10^ They generally imply a narrow definition of ‘medical’ focused on biological or organic factors and overlooking social and psychological factors. For example, a patient who needs rehabilitation may be judged ‘medically fit for discharge’ when social, psychological and physical needs have not been addressed. In this study we focus on the use of such terms in the context of referrals and collaborative working between acute hospital specialties and liaison psychiatry. The attitudes towards, and interpretation of, these terms by general medical and psychiatric clinicians remain undocumented.

We investigated liaison psychiatry referral practices in England with two aims: (a) to assess the prevalence of delaying liaison psychiatry assessment until the patient has received ‘medical clearance’ in practice and policy in general hospital settings and (b) to investigate how ‘medical clearance’ terms are defined and understood by relevant clinicians.

## Method

### Data collection

#### National analysis of trust referral policies

Freedom of information (FOI) requests were sent to all acute National Health Service (NHS) mental health trusts in England. Requested information included the type of liaison psychiatry service, hospital site(s), whether a referral policy was used, whether the policy detailed referral criteria related to ‘medical clearance’, and a copy of the policy. The list of contacted trusts and the FOI request template are given in Supplementary Items A and B, available at https://doi.org/10.1192/bjb.2023.43. Acute hospital trusts were contacted if they provided their own psychiatry services to in-patients. Initial enquires were sent in December 2019. Trusts that did not respond to at least three requests between December 2019 and March 2020 were excluded from the analysis.

#### National survey of liaison psychiatry

The 5th Survey of Liaison Psychiatry in England (LPSE-5) was conducted across all acute hospitals with emergency departments in England.^11^ The questions from LPSE-5 relevant to this analysis are given in Supplementary Item C. Data collection took place from June to December 2019.

#### National survey of referring junior doctors

An online survey was cascaded to junior doctors working in patient-facing specialties in acute hospitals across England via all Health Education England postgraduate deaneries and schools. Participants were invited to participate in a ‘survey about referrals to liaison psychiatry’. Data collection took place from June to August 2020. The survey asked about respondents’ experience of making referrals to liaison psychiatry. Survey items are detailed in Supplementary Item D.

### Analyses

A content analysis of survey responses and trust referral policies was performed using coding tags relating to ‘medical clearance’.^12^ This approach permitted quantification of different ‘medical clearance’ terms (e.g. ‘medically fit’, ‘medically fit for assessment’, ‘medically fit for discharge’), the context in which they were used (e.g. during referral for psychiatric assessment, or whether the terms were explicitly discouraged) and their perceived definitions.

Additional thematic analyses were performed for the survey responses.^13^ A thematic analysis was performed for the liaison psychiatry survey, to explore the working definitions of and rationalisations for using ‘medical clearance’ terms and to enable the junior doctor survey to explore the perceived barriers to successful referrals and doctors’ confidence using such terms.

### Ethics statement

Following consultation with the Health Research Authority, the project was deemed to be a service evaluation and therefore did not require review by an NHS Research Ethics Committee.

## Results

### National analysis of trust referral policies

Of the 56 contacted trusts, 54 (96%) responded. A single trust-wide policy existed for 31 trusts, multiple site-specific policies existed for 13 trusts and 10 trusts reported having no policy; 38 trusts shared their trust-wide or site-specific policies, providing 48 policies for analysis.

#### Prevalence and definitions of ‘medical clearance’ terms

The proportion of policies that used ‘medical clearance’ terms is outlined in Table 1. Sixty per cent of policies used the terms ‘fit for assessment’ or ‘fit for interview’, which is consistent with good practice. Of the six trusts with policies that were not shared, three reported using ‘medically fit for assessment’ as a referral criterion and three denied doing so.

**Table 1:** The use of ‘medical clearance’ terminology in local National Health Service trust policies and as reported in the liaison psychiatry and junior doctor surveys

Data source and terms	Frequency
**Trust referral policies (*n* = 48)**	
Of total policies, *n* (%)	
Used one or more ‘medical clearance’ term^a^	36 (75)
‘Medically fit for assessment’	20 (42)
‘Medically fit for interview’	9 (19)
‘Medically fit’	9 (19)
‘Medically fit for discharge’	8 (17)
Other related terms^b^	5 (10)
Of policies using such terms, *n* (%)	
Used as referral criteria (i.e. to delay or decline psychiatric assessment)^c^	27 (75)
Explicitly not used as referral criteria^d^	13 (36)
Of policies using terms as a referral criterion, *n* (%)	
Descriptive definition provided	6 (22)
Illustrative example provided	3 (11)
Neither provided	18 (67)
**Liaison psychiatry survey (*n* = 173)**	
Of total respondents, *n* (%)	
Used one or more ‘medical clearance’ term^e^	136 (79)
‘Medically fit for assessment’	51 (29)
‘Medically fit’	44 (25)
‘Medically fit for discharge’	40 (23)
‘Medically fit for interview’	9 (5)
Other related terms^f^	26 (15)
Of respondents using such terms, *n* (%)	
Used as referral criteria (i.e. to delay or decline psychiatric assessment)	111 (82)
‘Medically fit for assessment’	49 (44)
‘Medically fit for discharge’	18 (16)
‘Medically fit’	15 (14)
‘Medically fit for interview’	9 (8)
Other related terms^g^	20 (18)
**Junior doctor survey (*n* = 486)**	
Of total respondents, *n* (%)	
Been told patients could not be seen due to ‘medical clearance’ terminology	412 (85)
This has delayed patients being seen	269 (55)
This has prevented patients being seen	242 (50)
Of respondents encountering such terms, *n* (%)	
Term used:	
‘Medically fit’	183 (44)
‘Medically cleared’	183 (44)
‘Medically fit for discharge’	55 (13)
Other related terms^h^	58 (14)
Interpretation in-keeping with:	
Treatment completion/ready for discharge	266 (65)
Exclusion of organic aetiologies	160 (39)
Fitness for assessment	27 (7)
Did not know	19 (5)

a. Thirteen policies used multiple terms within the same policy.

b. Other terms included: ‘medically cleared’ (2), ‘able to be assessed’ (1), ‘not requiring urgent medical treatment’ (1) and ‘medically stable’ (1).

c. Nine policies used different terms interchangeably, as referral criteria, in the same policy.

d. Four policies used ‘medically fit for assessment’ or ‘medically fit for interview’ as a referral criterion, but explicitly advised against using ‘medically fit for discharge’.

e. Refers to the number of respondents citing a ‘medical clearance’ term in their survey response.

f. Other terms included: ‘medical clearance’ (12), ‘medically stable’ (5), ‘medically well’ (3), ‘[physically] able to engage’ (2), ‘medically optimised’ (1), ‘medically reviewed’ (1), ‘all physical investigations & treatment completed’ (1) and ‘able to hold a logical conversation and have capacity to make an informed decision’ (1).

g. Other terms used included: medical clearance’ (6), ‘medically stable’ (5), ‘medically well’ (3), ‘[physically] able to engage’ (2), ‘medically optimised’ (1), ‘medically reviewed’ (1), ‘all physical investigations & treatment completed’ (1) and ‘able to hold a logical conversation and have capacity to make an informed decision’ (1).

h. Other terms included: ‘organic/physical aetiology ruled out’ (31), ‘no ongoing medical issues’ (8), ‘medically optimised’ (7), ‘medical treatment ongoing’ (6), ‘medically stable’ (5), ‘medically ready’ (4), ‘medically fit for assessment’ (4), ‘medically unsafe’ (1) and ‘medically unsuitable’ (1).

Definitions of ‘medical clearance’ terminology were rarely provided and varied considerably (Table 2)

**Table 2:** Example quotes of how terms related to ‘medical clearance’ are used and defined in local trust policies

Term	Descriptive definitions	Illustrative examples
Medically fit	‘A patient who is fit to be sent home and would be discharged if there were no mental health concerns’ ‘Received all recommended [medical] treatment’	
Medically fit for assessment	‘(i) Not intoxicated [and] (ii) not needing urgent medical treatment’ ‘No physical health concerns, EWS < 2’ ‘No organic cause for symptoms identified [following medical history examination and investigations] and delirium tremens excluded’ ‘Is able to communicate, present on the ward’ ‘For a patient to be fit for assessment the following guidelines should be followed: (i) patient is not suffering the effects of a drug overdose or its treatment so that they can undertake a possible 2-hour assessment without falling asleep, vomiting or suffering the effects of the overdose in other ways that may have a detrimental effect on the assessment, (ii) patient is not in a toxic, confusional state caused by drugs, (iii) patient is not under undue influence of alcohol or drugs (prescribed or recreational), (iv) patient will ideally have had all invasive treatments completed so that the clinical picture is not distorted by these later, (v) if the patient is usually ambulant then it should be expected that they are ambulant enough to be seen away from the bed space to ensure privacy, confidentiality and dignity is upheld’	‘[Being medically unfit for assessment] will be the case if someone is under care on an intensive care unit’ ‘Maintaining a level of clear consciousness that would allow meaningful assessment of their mental state to occur’ ‘[When a patient is intoxicated] a clinical decision [should be] made as to the impact that the level of intoxication is having on the person's behaviour, speech and cognition’
Medically fit for interview	‘Fitness for interview is defined as; the patient is able to mobilise and hold a coherent and sustained conversation and is not displaying undue physical symptoms. Treatment may be on-going but discharge is imminent’	

EWS, early warning score (on the National Early Warning Score tool).

### National survey of liaison psychiatry

All 173 (100%) acute hospitals responded.

#### Clinicians’ accounts of ‘medical clearance’ terms

The proportion of respondents mentioning ‘medical clearance’ terms in relation to referral criteria is outlined in Table 1. Clinicians’ working definitions of ‘medical clearance’ terms are quoted in Table 3.

**Table 3:** Quotes of how terms relating to ‘medical clearance’ are used and defined by liaison psychiatry clinicians

Term	Descriptive definitions	Illustrative examples
Medically fit for assessment	‘Physical impairment is not impacting on mental health’ ‘In a position to do an assessment, not intoxicated’ ‘We utilize NEWS scores to determine someone's fitness’ ‘We ask if the staff would be happy for the person to get behind the wheel of a car’	‘Not drowsy or vomiting’ ‘Alert and not intoxicated’ ‘e.g. [not] intoxicated or altered states of consciousness’ ‘Be able to talk, not delirious’ ‘e.g. extubated’
Medically fit for interview		‘Conscious and able to speak’ ‘Able to participate fully in verbal assessment’ ‘Not intoxicated by alcohol or drugs and have capacity unless psychotic of manic’
[physically] able to engage		‘Patient may be un-assessable due to intoxication, sedation or if in receipt of extensive medical interventions’
Medically fit	‘Require physical cause for presentation to be ruled out’	
Medically cleared	‘Any medical condition which may be influencing presentation to be ruled out’ ‘Patients will not be seen if they require investigation or treatment’	‘If there [is] evidence of infection for example, we would defer assessment until bloods had been checked’ ‘Referrals are refused if the patient is intoxicated or delirious’
Medically stable		‘e.g. not under the influence of alcohol’
Medically well	‘i.e. not acutely unwell or symptomatic’	

NEWS, National Early Warning Score tool.

Six themes were generated: awake and coherent, medical investigations, comorbid conditions, intentional delay, workload management and interprofessional differences.

##### Awake and coherent

Many liaison psychiatry services would only accept a referral if a patient was able to respond coherently to a clinical interview. Referrals were not accepted for people who may be intoxicated with alcohol or drugs or who were unconscious. Some respondents referred to a requirement for patients to be ‘conscious and able to speak’, ‘have capacity’, be ‘able to participate fully’ or be ‘able to hold a logical conversation’. One respondent reported asking referring clinicians whether they would be ‘happy for the person to get behind the wheel of a car’ as a criterion for accepting referrals.

##### Medical investigations

Some respondents required that ‘physical causes for a presentation’ or ‘any medical condition influencing presentation’ were ‘ruled out’ prior to psychiatric referral.

##### Comorbid conditions

Some respondents required that patients were not ‘acutely unwell or symptomatic’ prior to psychiatric assessment. Some respondents reported concerns that physical symptoms may be ‘impacting on mental health’, whereas others appeared more concerned with physiology beyond symptomology. Some respondents reported using physical health scales to establish ‘medical clearance’, for example ‘we utilize NEWS [the National Early Warning Score tool] scores to determine someone's fitness’. For others, ‘medical clearance’ hinged on patients not being ‘in receipt of extensive medical interventions’ prior to psychiatric assessment. There was inconsistency relating to delirium. Some respondents referred to ‘altered states of consciousness’, ‘delirious’ or ‘evidence of infection’ as reasons to delay or decline referrals, whereas others explicitly accepted referrals for patients experiencing delirium.

##### Intentional delay

Many respondents reported delaying assessment until ‘medical clearance’ for subgroups of patients, typically those who had self-harmed. One respondent expressed the view that delaying liaison psychiatry response might be helpful: ‘we sometimes wait for people to be medically able to be discharged when we know an assessment immediately prior to discharge is more therapeutic for them’. Another respondent expressed the opinion that seeing patients ‘when they are medically stable can lead to [an] increase in risk behaviour [if] the outcome [of psychiatric assessment] is not what the service user wants’.

##### Workload management

Respondents described managing workload by delaying response for ‘medical clearance’: ‘staffing levels often mean we may wait for, or prioritise, patients that are medically fit’ and ‘we usually see service users at the point of discharge in order to make a plan as there is little time with such a small team to revisit service users to confirm plans’.

##### Interprofessional differences

A minority of respondents described interprofessional differences of view, and associated dissatisfaction. One respondent stated that patients are ‘declared “medically fit” when no investigations or even basic observations have been done as the emergency department fears a delay’. In contrast, another respondent who did not require ‘medical clearance’ before liaison psychiatry response reported a ‘misconception amongst [referring] junior doctors that we will reject earlier referrals [due to a perceived lack of medical fitness]’, which led to ‘frustrating’ delays.

### National survey of referring junior doctors

We received 516 responses to the junior doctor survey, of which 486 (94%) were complete. The response rate is unknown because we do not know how many junior doctors saw the mailing. Demographic details of the respondents are in Supplementary Item E.

#### Accounts of non-acceptance by liaison psychiatry

The most common reported barrier to successful referrals related to ‘medical clearance’. Two main themes were generated: medical investigations and comorbid conditions. Additional themes are described in Supplementary Item F.

##### Medical investigations

Respondents described ‘stringent requirements’ relating to patients’ physical health. This typically related to excluding organic aetiology, including resolution of borderline blood tests (urea, creatinine, sodium and C-reactive protein), neuroimaging (magnetic resonance imaging (MRI) and computed tomography (CT) of the head) and lumbar punctures being required before psychiatric referral. Disagreements over whether delirium was an indication for liaison psychiatry review were frequently reported. One respondent reported ‘[liaison psychiatry] commonly review the notes remotely and put the presentation down to delirium so won't review’.

##### Comorbid conditions

*Physical signs or symptoms*: Many respondents were unable to refer patients with comorbid physical conditions, irrespective of their perceived relevance: ‘my hospital sees a large number of patients with eating disorders and the liaison psychiatry service usually refuses to see them as they are not “medically fit”’ and ‘where a patient has a known psychiatric condition and physical health problem, we seem unable to deal with the psychiatric aspect of their presentation until they have had all other problems fully addressed’. Respondents reported that this practice was especially problematic when patients’ mental health influenced their physical health: ‘sometimes patients aren't able to become medically fit because of their [psychiatric] issues; i.e. they become dehydrated because they are not eating or drinking because of their depression’.

*Receipt of treatment*: Respondents frequently reported that psychiatry services required resolution of all medical treatment before reviewing patients: ‘if the patient is on intravenous treatment, liaison psychiatry would say they are not “medically fit”, even though there is nothing preventing them from being seen or assessed’. Others reported that referrals had been declined because of minor epistaxis, borderline hypokalaemia, the patient being admitted to an intensive care setting or having a catheter or cannula *in situ*.

*Comorbid alcohol or substance use*: Respondents reported substance misuse history as a common exclusion criterion: ‘if they have any history of drug or alcohol abuse, psychiatry won't see them’, ‘where drug and alcohol use are comorbid, this prevents referrals despite being an intrinsically psychiatric issue; e.g. alcohol use with depression or mania’ and ‘one patient had expressed thoughts of suicide and current severe low mood; I was informed by psych liaison that they would not come to see him due to his admission of excess alcohol intake’.

#### Prevalence and definitions of ‘medical clearance’ terms

The majority of respondents had been told that patients could not be seen by liaison psychiatry because of a criterion related to ‘medical clearance’ (Table 1). Respondents often interpreted these terms to mean completion of treatment or the exclusion of organic aetiologies (Table 1).

#### Confidence using ‘medical clearance’ terms

There was substantial variety in respondents’ confidence assessing ‘medical clearance’ criteria, and various themes were generated.

##### Conceptual vagueness

*Lack of meaning*: A number of respondents reported scepticism towards the concept of ‘medical clearance’, reporting they were ‘unsure what the term actually means’, that there was ‘no real definition of “medically fit”’, that it was ‘a false term which makes no medical sense’ and that the concept was ‘nebulous’ and ‘ambiguous’. The definition of ‘medical clearance’ was inconsistent within departments: ‘the criteria often seem to vary in definition with each liaison clinician’ and the ‘standards seem to differ for criteria depending on who is being referred to’. Others suggested a general discrepancy between medical and psychiatric teams: ‘the opinions on what constitutes medically fit may differ between us and psychiatrists’ and ‘my threshold for which is “medically unfit” is a significantly higher than that of my psychiatry colleagues’.

*Misleading*: Respondents also felt that the concept of completely ‘excluding’ organic pathology was unrealistic and a ‘misnomer’: one respondent wrote ‘I don't think anyone can ever 100% “rule out” organic pathology and don't think that is a helpful way of viewing things’, and another noted ‘it's difficult and dangerous to ever be 100% certain the patient doesn't have a medical problem as a cause of their symptoms’. Referring doctors often reported that the decision to refer to psychiatry should be made ‘on balance of probabilities’ rather than ‘completely excluding [organic aetiology]’, leading some respondents to have had purposefully circumvented referral criteria: ‘it's technically impossible to meet the criteria, so we just wing it’. Respondents also reported feeling concerned that terms related to ‘medical clearance’ may misrepresent patients’ medical comorbidities, irrespective of aetiology: ‘the term is meaningless; someone with several stable but serious chronic health conditions can be “medically cleared” and from that point they may be assumed by psychiatry staff not to have any physical health needs at all’ and ‘[it] can be difficult if they have medical comorbidities; sometimes it feels like I am expected to promise that someone will not need any medical follow-up or investigations after I “medically clear” them’.

*Lack of expertise*: A number of respondents reported feeling unqualified to exclude rarer causes of psychiatric symptomatology: ‘all the pathologies which can affect the brain can be quite daunting’ and ‘I'm uncomfortable with the idea of “medical clearance” given my lack of specialist neuropsychiatric training’. Respondents often reported that they would benefit from psychiatric input when formulating differential diagnoses, rather than requiring ‘medical clearance’ before review: ‘if a psychiatric [presentation] is indeed due to some organic cause then the input from a psychiatrist might be helpful in the diagnostic pathway’, ‘I'm unable to “exclude” organic causes all the time, [but] these patients would still benefit from liaison psychiatry input’ and ‘there is often lingering doubt and it would be helpful if there was more of a collaborative approach rather than a black and white, medical vs psychiatric diagnosis’. Despite this, respondents often felt unable to seek such collaboration: ‘if there is a possible medical cause I can't refer to psychiatry’. A minority of respondents cited a lack of previous training in assessing ‘medical clearance’: ‘no one has ever taught on this’ and ‘there is no clear guidance’.

*Lack of experience*: Many respondents reported they were too junior to make decisions regarding ‘medical clearance’, often reporting that it should be a consultant-led decision: ‘as an FY1 [Foundation Year 1 doctor] I cannot discharge patients or say they are medically fit’ and ‘it is the responsibility of the treating consultant to [decide] if patients are medically fit’; others delayed psychiatric referral to first seek senior advice: ‘I will usually involve the registrar or the consultant; if it's out of hours, I will keep the patient till the following morning’.

*Practical difficulties of clinical assessment*: Some respondents highlighted the difficulty of assessing the physical health of acutely distressed patients: ‘some patients are hard to “medically clear” as they are not amenable to examination/investigations’.

*Fitness-for-discharge judgements*: Among those who reported feeling confident assessing ‘medical clearance’, it was nearly always interpreted as fitness for discharge: ‘I'm very confident, given they are essentially the same criteria we would use for discharge home’. Respondents also cited local experience as the source of their confidence: ‘after working in a hospital you gain a reasonable understanding about what the local liaison team expects’ and one respondent credited local support for referring junior doctors: ‘[I'm] relatively confident as there has been a recent local initiative to provide guidance [using] a pro-forma’.

## Discussion

In three parallel investigations, we assessed the prevalence, terminology and definitions associated with the use of ‘medical clearance’ prior to liaison psychiatric assessment in local NHS trust policies and practice.

Use of ‘medical clearance’ terminology is widespread and lacks conceptual clarity. The term signifies anything from patients being awake and talking to the absence of comorbid physical symptoms or cognitive impairment. We identified common themes across the junior doctor and liaison psychiatry surveys. These included ‘medical clearance’ terminology being used to require medical aetiology to be ‘excluded’ and requirements that patients had no comorbid physical illness or ongoing medical intervention prior to referral. Liaison psychiatry services commonly reported using ‘medical clearance’ to ensure patients were awake and talking, although this theme was not reported by junior doctors. Most junior doctor respondents (55%) reported that ‘medical clearance’ terminology has delayed patient care, and half (50%) reported it has prevented patients being seen by a member of the liaison psychiatry team.

### Interpretation within a human factors framework

Our findings can be considered in the human factors framework of work-as-imagined, work-as-prescribed, work-as-disclosed and work-as-done.^14^ Work-as-imagined refers to the mental models held about the work we or others do, work-as-prescribed is the formal description or specification of work (for example in policy or procedures), work-as-disclosed is the work that staff describe doing, and work-as-done is the work activities that are actually conducted. There is widespread discrepancy between work-as-prescribed in UK good practice guidance and work-as-prescribed in local policy.^6,7,15–17^ We found similar discrepancies when considering work-as-disclosed in the two surveys. Recent consensus guidance urged clinicians to ‘eradicate unhelpful terms such as “medically fit” or “medical clearance” in favour of providing timely, appropriate mental health assistance and side by side working’ (p. 4).^6^ Our findings suggest that this aspiration is not yet achieved. There is a similar discrepancy between work-as-imagined and work-as-disclosed: although liaison psychiatry is commonly seen as holding subspecialist expertise in the complex interface between medical conditions and mental disorders, referrals are rejected on grounds of medical complexity.^18^ Particularly concerning examples included declining referrals for patients in receipt of minor medical treatments or those admitted to intensive care, and using physical mobility and arbitrary thresholds related to daily functioning (including fitness to drive) as exclusion criteria. Likewise, the practice of delaying assessment until the point of hospital discharge in case the outcome led to an ‘increase in risk behaviour’ suggests that timing of mental healthcare may be related to system priorities and coercive practice rather than patient need. Notably, no survey respondent considered patients’ preference regarding the timing and purpose of liaison psychiatry review.

At least three of our themes may account for the persistence of these working practices. Interprofessional difficulties may indicate a lack of trust or communication between specialties, leading to concerns that early assistance from psychiatry will be interpreted as taking over care and lead to a withdrawal of medical input. Lack of expertise in the management of concurrent medical conditions and mental disorders may also result in avoidance or deferral. Delaying responses to referrals may also be considered as an approach to workload management in the context of widespread understaffing of liaison psychiatry.

### Future research

Future work to improve clinical practice should identify and address these maintaining factors. Investment in adequate staffing levels, alongside interventions for working culture and practice, may be necessary. Workforce development must include an increase in both staff numbers and development of clinical skills. Existing good practice standards should be effectively communicated and peer accreditation networks may have a role in promoting the uptake of practice standards.^19^

### Strengths and limitations

Our findings are in keeping with previous literature.^5,10,15^ This is the largest study on the use of ‘medical clearance’ to date and it benefitted from triangulation of findings from three different approaches. Importantly, exploring patients’ perspectives on ‘medical clearance’ was beyond the scope of this study and is a worthy area of future investigation. Previous work suggests that patients perceive the process of ‘medical clearance’ to be a frustrating and generally negative experience.^20^

Although the study was confined to England and the policy analysis was limited by non-response of a number of trusts, our analyses featured good geographical diversity. Our findings may well be generalisable to the whole UK. The survey of liaison psychiatry services relied on a single informant for each site. Although self-report surveys may be subject to social desirability bias, the responses describing considerable discrepancies in relation to good practice guidance suggests this was not a serious problem. The junior doctor survey may have been subject to self-selection bias, for example for respondents with particularly strong or negative views on the topic. However, the responses were consistent with responses from the liaison psychiatry survey, which had a 100% response rate. The use of examples in the junior doctor survey may have primed respondents towards sharing accounts of negative practice, although their use was deemed necessary to clarify the extent to which patient care was delayed or prevented by ‘medical clearance’ terminology.

Data collection on trust referral policies and the liaison psychiatry survey were conducted prior to the COVID-19 pandemic. Changes in service configuration and working practices, such as the development of non-co-located mental health emergency centres, may have had an adverse impact on the provision of joint care and may be an important area of future investigation.

In conclusion, our national service evaluation found common use of ‘medical clearance' terminology in both practice and policy. We identified evidence that patient care is delayed and sometimes prevented by such practices. ‘Medical clearance’ terms lacked meaningful definitions, were used inconsistently and were poorly understood by referring junior doctors. There is a discrepancy between UK good practice guidance, local policies and reported working practices that warrants further investigation and action. Further research priorities include a comparative analysis of local policies against UK guidance, ethnographic study of work-as-done to investigate the impact on patients, and evaluation of interventions to improve practice.

## About the authors

**George Gillett** is an NIHR Academic Clinical Fellow at the Institute of Psychiatry, Psychology and Neuroscience, King's College London, London, UK and formerly an NIHR Academic Foundation Programme doctor at the Department of Psychiatry, University of Oxford, Oxford, UK. **Sophie Westwood** is a Research Assistant in the Faculty of Health at the University of Plymouth, Plymouth, UK. **Alex B. Thomson** is a Consultant Liaison Psychiatrist with Central and North West London NHS Foundation Trust, working in the Department of Psychological Medicine at Northwick Park Hospital, London, UK. **William Lee** is a Consultant Liaison Psychiatrist with Cornwall Partnership NHS Foundation Trust, Bodmin, UK

## Supporting information

Gillett et al. supplementary material 1 Gillett et al. supplementary material

Gillett et al. supplementary material 2 Gillett et al. supplementary material

## Data Availability

The data are available from the corresponding author, G.G., on reasonable request.
