# Supplementary material for: ‘Medical clearance’ and referral to liaison psychiatry: a national service evaluation
Source: BJPsych Bull. 2024 Jun;48(3):151–8. doi: 10.1192/bjb.2023.43 (PMC11134037; doi:10.1192/bjb.2023.43)
Supplement: Gillett et al. supplementary material 1 — Gillett et al. supplementary material [file S2056469423000438sup001.docx]

**Supplementary Material:**

Supplementary Item A: List of Trusts contacted with Freedom of Information Requests

The list of Trusts was based on internet searches, NHS England data and an FOI request to the CQC.

*List of acute mental health trusts:*

Avon and Wiltshire Mental Health Partnership NHS Trust

Barnet, Enfield and Haringey Mental Health NHS Trust

Berkshire Healthcare NHS Foundation Trust

Birmingham and Solihull Mental Health NHS Foundation Trust

Black Country Partnership NHS Foundation Trust

Bradford District Care NHS Foundation Trust

Cambridgeshire and Peterborough NHS Foundation Trust

Camden and Islington NHS Foundation Trust

Central and North West London NHS Foundation Trust

Cheshire and Wirral Partnership NHS Foundation Trust

Cornwall Partnership NHS Foundation Trust

Coventry and Warwickshire Partnership NHS Trust

Cumbria, Northumberland, Tyne and Wear NHS Foundation Trust

Derbyshire Healthcare NHS Foundation Trust

Devon Partnership NHS Trust

Dorset Healthcare University NHS Foundation Trust

Dudley and Walsall Mental Health Partnership NHS Trust

East London NHS Foundation Trust

Essex Partnership University NHS Foundation Trust

Gloucestershire Health & Care NHS Foundation Trust

Greater Manchester Mental Health NHS Foundation Trust

Hertfordshire Partnership University NHS Foundation Trust

Humber Teaching NHS Foundation Trust

Kent and Medway NHS and Social Care Partnership Trust

Lancashire Care NHS Foundation Trust

Leeds and York Partnership NHS Foundation Trust

Leicestershire Partnership NHS Trust

Lincolnshire Partnership NHS Foundation Trust

Mersey Care NHS Foundation Trust

Midlands Partnership NHS Foundation Trust

Norfolk and Suffolk NHS Foundation Trust

North East London NHS Foundation Trust

North West Boroughs Healthcare NHS Foundation Trust

North Staffordshire Combined Healthcare NHS Trust

Northamptonshire Healthcare NHS Foundation Trust

Nottinghamshire Healthcare NHS Foundation Trust

Oxford Health NHS Foundation Trust

Oxleas NHS Foundation Trust

Pennine Care NHS Foundation Trust

Rotherham Doncaster and South Humber NHS Foundation Trust

Sheffield Health and Social Care NHS Foundation Trust

Somerset Partnership NHS Foundation Trust

South London and Maudsley NHS Foundation Trust

South West London and St George's Mental Health NHS Trust

South West Yorkshire Partnership NHS Foundation Trust

Southern Health NHS Foundation Trust

Surrey and Borders Partnership NHS Foundation Trust

Sussex Partnership NHS Foundation Trust

Tees, Esk and Wear Valleys NHS Foundation Trust

West London Mental Health NHS Trust

Worcestershire Health and Care NHS Trust

*List of general trusts providing inpatient liaison psychological medicine services:*

Gateshead Health NHS Foundation Trust

Isle of Wight NHS Trust

North Bristol NHS Trust

Northumbria Healthcare NHS Foundation Trust

Oxford University Hospitals NHS Foundation Trust

Supplementary Item B: FOI Request Template, sent to trusts

FOI: Liaison Psychiatry Referral Policy

Dear [Name of Mental Health Trust],

We are a group of clinicians & researchers carrying out a national evaluation of liaison psychiatry service provision in NHS hospitals across England. As part of this project, we are submitting FOI requests to all NHS Mental Health Trusts to ask about local policy and practice. We kindly request the following information as per the FOI Act 2000.

Our enquiries relate to the provision of liaison psychiatry services in adult general hospitals, including patients in inpatient wards or A&E departments. This research is conducted as part of an Academic Foundation Programme research post. No specific grant was awarded for this project. All findings will be de-identified from Hospitals or Trusts before publication.

1. At which general hospital(s) does your Trust provide liaison psychiatry services?

For each of the above hospitals, please answer the questions below:

1. Is there a written policy about prioritising patients who have been referred to liaison psychiatry, or determining which referrals to accept? (If No, please move to Question 6).
2. If so, is this policy made available to all (psychiatry and non-psychiatry) clinicians working within the general hospital? If so, please specify where (e.g. Trust intranet)?
3. Please attach the policy document if possible. We aim to extract only certain information from the policy document, so if the complete policy document cannot be sent, please send the following information:
   1. Details on which locations this policy covers and which patients (i.e. inpatient ward/A&E/referrals) the policy covers.
   2. Details on any guidelines to prioritise or determine which referrals to accept. Specifically:
      1. Patients should be medically fit for discharge prior to review
      2. Patients should be medically fit for assessment prior to review
      3. No restriction applied; all patients are reviewed
      4. Any other methods used to prioritise patient referrals (please specify)
   3. Whether the policy states which professional group (e.g. psychiatrist, psychologist, nurse) should review the referral and/or assess the patient? If so, please specify.
   4. When was the policy last reviewed or updated?
4. When (if at all) was local practice last audited against this policy and what were the findings?
5. At your Trust, how do doctors refer patients to liaison psychiatry for review (e.g. by telephone, by electronic referral system, by email, by fax)?

Many thanks for your time. Please do not hesitate to get in touch by email if you have any questions or clarifications. We look forward to your reply.

Yours faithfully,

Dr George Gillett,

Academic Foundation Programme Doctor

Department of Psychiatry, University of Oxford

Dr William Lee,

Honorary Clinical Senior Lecturer, University of Exeter Medical School.

Consultant Liaison Psychiatrist, Devon Partnership NHS Trust.

Supplementary Item C: National survey of liaison psychiatry questions

i) Service referral criteria (incl. age range) from ED: (please be as specific as you can, and please specify here whether you always/sometimes/never wait for patients to be ‘medically fit for discharge’, or ‘medically cleared’ before seeing them)

ii) Service referral criteria (incl. age range) from wards: (please be as specific as you can, and please specify here whether you always/sometimes/never wait for patients to be ‘medically fit for discharge’, or ‘medically cleared’ before seeing them)

Supplementary Item D: Junior doctor survey questions

Dear Doctor,

**National Survey of doctors in postgraduate training about referrals to Liaison Psychiatry**

This survey is for doctors in postgraduate training, working in Acute Hospital posts (i.e. not psychiatry, GP or other community posts) in England. It takes about five minutes to complete and respondents will be entered into a prize draw to win a £50 Amazon voucher.

This survey is part of a national service evaluation project led by clinical academics at the University of Oxford and University of Exeter. The project assesses referral practices to liaison psychiatry services across England and is funded by NIHR. All responses are confidential.

Thank you for taking part.

For further information please contact george.gillett@medsci.ox.ac.uk.

Please answer all questions as fully as possible. Many thanks for your time.

1. What is your level of training?
   1. FiY1
   2. FY1
   3. FY2
   4. CT1/ST1
   5. CT2/ST2
   6. CT3/ST3
   7. ST4
   8. ST5
   9. ST6+
   10. Other (please state)
2. What is your age & gender?
3. What specialty are you training in & at which acute hospital are you currently working? (If you are not working in a specialty post, please describe your post, e.g. Foundation Year 2)
4. Of the patients for whom you care and you think might benefit from liaison psychiatry input, what percentage are successfully referred to, accepted by, and then seen by liaison psychiatry?
5. If this is not 100%, what do you think prevents patients being referred to, accepted by, and seen by the liaison psychiatry team?
6. In your current hospital, have you ever been told by a liaison psychiatry professional that a patient cannot be seen, or cannot be seen yet, because of a criterion related to their ‘medical fitness’? (eg ‘not medically fit’, ‘not medically fit for discharge’, ‘not medically cleared’ etc?) (Note: This question is not about whether a patient is fit for psychiatric interview.)
   1. Yes – this has prevented patients being seen
   2. Yes – this has delayed patients been seen
   3. No
7. If yes, please name the term or terms used. (eg “Medically cleared”)
8. Please summarise your understanding of what that term means or what those terms mean.
9. Please describe how confident you feel in your ability to assess whether patients meet the criteria associated with this term or those terms, and why.
10. To be entered into the prize draw, please enter your email address.

Supplementary Item E: Demographic details of respondents to junior doctor survey

Specialty data includes respondents training at CT1 and above only, since FY doctors are not enrolled on a specialty training programme.

| **Age** |  |
| --- | --- |
| Mean (SD) | 30.40 (4.60) |
| **Gender**; N (%) |  |
| Male | 183 (37.65%) |
| Female | 287 (59.05%) |
| Non-binary | 2 (0.41%) |
| Prefer not to say | 14 (2.88%) |
| **Grade**; N (%) |  |
| FiY1 | 5 (1.03%) |
| FY1 | 79 (16.26%) |
| FY2 | 86 (17.70%) |
| CT1/ST1 | 57 (11.73%) |
| CT2/ST2 | 55 (11.32%) |
| CT3/ST3 | 43 (8.85%) |
| ST4 | 53 (10.91%) |
| ST5 | 43 (8.85%) |
| ST6+ | 65 (13.37%) |
| **Specialty**; N (%) |  |
| Anaesthetics/ICU | 45 (14.24%) |
| ACCS | 3 (0.95%) |
| Emergency Medicine | 33 (10.44%) |
| Core/Internal Medicine | 65 (20.57%) |
| Cardiology | 4 (1.27%) |
| Endocrinology | 2 (0.63%) |
| Haematology | 1 (0.32%) |
| Gastroenterology | 8 (2.53%) |
| Geriatric Medicine | 15 (4.75%) |
| Infectious Diseases | 5 (1.58%) |
| Neurology | 5 (1.58%) |
| Oncology | 11 (3.48%) |
| Palliative Medicine | 3 (0.95%) |
| Respiratory | 9 (2.85%) |
| Rheumatology | 7 (2.22%) |
| Renal Medicine | 7 (2.22%) |
| Core Surgery | 9 (2.85%) |
| ENT | 5 (1.58%) |
| General Surgery | 10 (3.16%) |
| Neurosurgery | 1 (0.32%) |
| Obstetrics & Gynaecology | 20 (6.33%) |
| Oral & Maxillofacial Surgery | 3 (0.95%) |
| Plastic Surgery | 3 (0.95%) |
| Trauma & Orthopaedics | 13 (4.12%) |
| Urology | 3 (0.95%) |
| Vascular Surgery | 1 (0.32%) |
| Paediatrics | 23 (7.28%) |
| Paediatric Surgery | 2 (0.63%) |

Supplementary Item F: Supplementary results; junior doctor survey

*Barriers to successful liaison psychiatry referral:*

*Recurrent attendances*

Respondents highlighted that referrals were particularly difficult for specific groups of patients, such as patients termed “recurrent attenders”. “If they are a recurrent frequent attendee to AE with a personality disorder often psych won’t see them”, “often patients who attend multiple times are ignored” and “patients are referred but not seen if mental health issues are longstanding rather than acute” respondents noted. The rationale often appeared to be that such patients had been recently assessed or were already under the care of crisis services, although on other occasions respondents reported being told psychiatric input wasn’t required due to patients’ presentations being “behavioural”.

*Risk assessment*

A lack of perceived risk was also cited as a common reason for referral rejection. “Mostly [rejection] seems to occur when the patient has mental health issues and are keen for psychiatric input themselves, but the risk is not deemed particularly high” noted one respondent. A substantial number of respondents replied in-keeping with “liaison psychiatry only review patients who have active suicidal ideation” and “patients can only be seen if pose a significant suicide risk”. Perhaps allied to that, one respondent noted their difficult getting input for patients with functional disorders or somatisation; “these patients are classically seen by liaison psychiatry teams as ‘not our problem’.” Other respondents reported that psychiatry teams often declined to see patients, stating that they could be followed-up by community services instead; “they often feel that the patient can be seen by community services without seeing the patient”.

*Registration with primary care*

A small minority of respondents had been told that patients could not be seen because they weren’t registered with a general practitioner; “he doesn’t have a GP therefore we can’t see him”.

*Recognition of need for psychiatric support*

A minority of respondents recognised factors associated with the medical team as barriers for successful referral. One respondent noted a lack of “recognition by the medical team that they need psychiatric input” while another noted that “the psychiatric elements of the history are not always taken by the medical admitting doctors, and not explored during ward rounds a lot of the time”, due to “medical problems being prioritised and deemed to be of primary concern”. Respondents noted that this led to “late involvement of psychiatry by which time patient may have been discharged”.

*Practical issues with referral*

Practical issues with the referral system were commonly reported as barriers to successful referral. Respondents reported unclear referral systems, not knowing who to contact, having to fax referrals or use paper forms which would often get lost. Respondents cited frustration at “pager systems which makes referrals very difficult” or “referring to administrative staff who pass on details [to psychiatry team]. Often they are very protocol driven and won’t adjust to nuance; it’s very difficult to speak to a psychiatrist if there are concerns”. Some respondents identified location as a barrier to referral; “the psychiatry team is at a different hospital, advice is usually given over the phone” and “psychiatry is in a separate building, not in the main hospital”.

*Service pressures*

A number of respondents speculated on more general themes contributing to referrals being refused. Lack of resources and capacity was identified by a many respondents. “Psychiatry are firefighting and have to triage cases because of their unmanageable workload”, “the mental health team are too busy to see all its patients” and “in every hospital I have worked, [liaison psychiatry] have been understaffed” were representative of a substantial number of responses. A number of respondents noted this was particularly problematic at night and weekends; “out of hours it feels like a car crash, admitting patients with self-harm for the weekend because the multi-site on call team cannot see them”. Respondents noted that service pressures also led to patients refusing psychiatry input; “psychiatry cannot come [immediately] because of understaffing and then the patient self-discharges in the interim”.

*Training among mental health clinicians*

Others suggested a perceived lack of medical experience among psychiatry staff may motivate the practice of delaying until ‘medical clearance’; “psychiatry staff are not comfortable dealing with what one might say are minor medical problems”. Respondents occasionally suggested issues relating to ‘medical clearance’ were unique to referring to psychiatry; “I’ve not come across another specialty that behaves this way”. Finally, respondents frequently reported a belief that ‘medical clearance’ terminology negatively impacted patient care, most overtly when leading to patients being perhaps unnecessarily sedated; “we often run out of ideas and end up re-intubating patients”.
